# Progress in Anæsthetics

**Published:** 1898-09-24

**Authors:** 


### Progress in Anæsthetics.

Chloroform as an anæsthetic still gives rise to considerable discussion, not as to its potency, but rather the danger in its administration. Whether it is dangerous under all circumstances, or only when carelessly or unskilfully administered, is a question which Waller does not answer positively.[1] He goes so far as to say that "chloroform administered for purposes of minor surgery is, *prima facie*, an unjustifiable event," and he believes that "the risk in its use for major operations may be considerably diminished by administration with a uniform percentage of chloroform vapour and air." "Quantity," he considers, "is an all-important factor in anæsthesia by chloroform, and in death by chloroform." These opinions are based upon the results of experiments performed upon a nerve, which was anæsthetised with different percentages of a mixture of chloroform vapour and air. Briefly, the experiments show that anæsthetisation with 4 per cent. is safe, with 5 per cent. the nerve is properly anæsthetised, with 6 per cent. dangerous.

It is a curious coincidence that Waller's results should have so closely corresponded with Snow's experiments performed nearly forty years ago, who found that the inhalation of a 5 per cent. mixture of chloroform vapour and air was sufficient to anæsthetise a human being.

Working upon Snow's dicta that 18 minims of pure chloroform circulating in the blood of a man produces surgical anæsthesia, and that 36 minims arrests the function of respiration, Waller finds that the amount of chloroform vapour in the lymph and blood in anæsthesia should not exceed 2 per cent., and therefore the lethal percentage would be 4. Snow's limit of 5 per cent. inhalation is dangerously high, and is not intended for continuous inhalation for any length of time; 1·5 per cent. is sufficient for this. From an analysis of a dozen long operations published by Hatch,[2] the average percentage of chloroform vapour was as low as ·85, the average amount of chloroform used per minute being 3·7 minims. If so little chloroform is needed to maintain a state of surgical anæsthesia, these writers are of opinion that by the open method of administration an overdose can very easily be given, and they strongly advocate a more scientific and graduated method of using this anæsthetic.

That the open method in Syme's principle is remarkably successful in some hands is strikingly illustrated by Surgeon-Lieutenant-Colonel Lawrie's return of chloroform administration at the Afzulgung Hospital during April, 1898[3]. The anæsthetic was administered by a third year student, and very successfully, considering that the average time to produce anæsthesia in children under five was 1′ 15″, in adults 3′ 36″. Lawrie follows Syme's method in "keeping the patients' breathing regular under all circumstances, and in entirely disregarding the heart and circulation as factors in the administration." By means of a diagram of the blood pressure under chloroform, the writer demonstrates very clearly the levels at which (*a*) anæsthesia should be maintained, (*b*) the breathing stops, (*c*) the pulse commences to fail, (*d*) the heart stops. The anæsthesia level is well above the region of danger. Were it universally accepted that the respiration ceases before the pulse commences to fail, the administration of chloroform ought to be an easy task, but in opposition to Lawrie's views, again repeated,[3] that chloroform has no direct action on the heart, we have the experiments of McWilliams, Gaskell, Shore, Hare, and Thornton. These observers found that failure of the heart took place during primary anæsthetisation in several animals, while the respiration contiuued unaffected. On rapidly opening the thorax the heart was found to be in a state of paralytic dilatation. Naturally they deduce that chloroform has a direct action upon the heart.[2]

Lawrie attributes the apparent early heart failure to administering the anæsthetic with too little admixture of air, and Dr. Bunford[3] discovered that "these sudden falls of blood pressure are due to nervous agency through the vagus, which, when overdosing is threatened, temporarily stops or slows the heart as a safeguard against the over-rapid conveyance of the anæsthetic to the brain."

Waller pertinently remarks that to save life, the end is more likely to be attained by attending to the "arithmetic" of chloroform vapour than by discussing whether chloroform produces primary cardiac syncope, or kills by the heart or by the respiratory centre.[1]

However great the difference may be on the foregoing subject, anæsthetists are all agreed that chloroform syncope is more likely to occur in the early stages of its administration. It is due to muscular spasm, struggling, and holding the breath; the strain upon the right heart is consequently increased, the venous system is congested, and the arterial tension lowered; the excess of $C.O_2$. in the blood at length stimulates the respiratory centre to give rise to deep inspirations; it is then that a large quantity of the anæsthetic is taken in and conveyed by the coronary arteries to the heart, with the result that that organ is thrown into a state of paralytic dilatation.

This, then, being the dangerous stage of chloroform administration, the practice advocated by Hewitt[4] of inducing anæsthesia by a safer anæsthetic, and then changing to chloroform, should be of great benefit, and diminish the number of fatal cases. He first anæsthetises with A.C.E. mixture, ether, or nitrous oxide gas followed by ether, and then continues the anæsthetisation with chloroform. The change, he urges, should be made before the commencement of the operation, and that at the time of the change the conjunctival reflex should be present; the latter point is of great importance to prevent too much of the more potent anæsthetic being "absorbed by the rapid respiration and brisk circulation which has been brought about by the ether." He claims that by this system the risk of pulmonary and bronchial complications is much diminished.

Burton[5] recommends a mixture of chloroform and ether in the proportion of 1 to 16. He contends that the amount of ether in the A.C.E. mixture is far too great, "it being calculated to blind the administrator to the cumulative deadening influence of the chloroform in the tissues, especially of the nerve centres." Also that A.C.E. administration in lengthy operations is followed by more severe and prolonged vomiting than after the above mentioned mixture.

Mr. W. Tyrrell[6] suggests a mixture of chloroform and ether by the addition of ether vapour from a second bottle during chloroform administration by Junker's apparatus.

Nitrous oxide gas and oxygen combined have, in Hewitt's hands,[4] proved a serviceable anæsthetic in surgical cases of some duration. On one occasion, for an amputation of the breast, this anæsthetic was administered for 35 minutes.

For dental purposes the administration of nitrous oxide gas may be prolonged for periods to five minutes by means of the recent suggestions of Mr. Coleman and Mr. Coxon, the former using a mask fitting accurately over the nose; the latter a bent tube introduced into the mouth, and held a short distance from the uvula.[7], [8]

The selection of an anæsthetic, in patients the subjects of renal disease, is a matter of difficulty. In one case, recently recorded, acute pulmonary œdema followed the administration of ether[9]; in another the albumen was increased by the administration of chloroform.[9]

With the above two cases was also reported one in which A.C.E. administration gave rise to albuminuria and jaundice.[9]

Schleich's mixtures, which have been largely experimented with in New York, do not appear to come up to expectation. In theory these mixtures are safe, as by reducing the maximum evaporation of the narcotic to almost the temperature of the body, the amount of the anæsthetics eliminated during evaporation almost equals that absorbed during the previous inspiration.[10] But in practice they have not proved so satisfactory, and Meyer, who on a previous occasion strongly advocated them, has discovered in them an element of some danger.[11] Schleich used a mixture of ether, chloroform, and petrolic ether, and it was supposed that the mixing of these substances was a true solution, in a chemical sense, and not a mere mixture of different ethereal substances. This has now been shown not to be so, and that they are merely mixtures and not solutions. Experiments on animals also show that the inhalation of petrolic ether is attended with the most dire results. Before, therefore, Schleich's mixtures can be used as general anæsthetics some substitute must be found for the petrolic ether, which will enter into chemical combination with the molecular combination of ether and chloroform, and thereby reduce the point of maximum evaporation.

In the treatment of emergencies under anæsthetics, Mr. Alexander Wilson[12] considers that "inflation and Silvester's method of artificial respiration are the most efficient, but faradisation of the phrenic nerves in old subjects with rigid chests, succeeds when other methods fail." In performing artificial respiration, Professor Schäfer considers the performance of expiration the more important. He also advocates the use of atropine before the administration of chloroform as a means of preventing arterial dilatation, and consequent fall of blood pressure. In his opinion the two drugs which promote the greatest contraction of the arterioles, and so would counteract the fall of blood pressure under chloroform, are nicotine and extract of suprarenal capsules. Dr. Silk regards strychnine as a valuable prophylactic; he also believes in oxygen in forced artificial respiration, and the actual kneading of the heart to empty it. Professor Hobday[13] considers hydrocyanic acid a useful antidote to chloroform, but Mr. Barnard[12] regards it as a dangerous drug.

[1] Brit. Med. Jour., April 23, 1898, p. 1057. [2] South African Med. Jour., April. 1898, p. 293 [3] Lancet, June 18, 1898, p. 1681 [4] Lancet, Feb. 19, 1898, p 485. [5] Lancet, April 30, 1898, p. 1534. [6] Clin. Jour., Jan. 26, 1898, p. 227. [7] Clin. Jour., May 25, 1898, p. 91. [8] Clin. Jour., June 1, 1898, p. 116. [9] Clin. Jour., June 8, 1898, p. 134. [10] Med. Rec., Jan. 8, 1898, p. 62. [11] Med. Rec., April 23, 1898, p. 607. [12] Lancet, Feb. 5, 1898, p. 369. [13] Lancet, Jan. 1, 1898, p. 27.

